# Testing the Sensitivity of Potential Panelists for Wine Taint Compounds Using a Simplified Sensory Strategy

**DOI:** 10.3390/foods7110176

**Published:** 2018-10-24

**Authors:** Marianne McKay, Florian F. Bauer, Valeria Panzeri, Astrid Buica

**Affiliations:** 1 Department of Viticulture and Oenology, Stellenbosch University, Stellenbosch 7600, South Africa; fb2@sun.ac.za (F.F.B); abuica@sun.ac.za (A.B.); 2 Institute for Grape and Wine Sciences, Stellenbosch University, Stellenbosch 7600, South Africa; panzeri@sun.ac.za

**Keywords:** odor detection threshold, wine, volatile phenol, 3-isobutyl-2-methoxypyrazine, 2,4,6-trichloroanisole

## Abstract

The odor detection threshold (ODT) of a compound is the lowest concentration at which individuals can reliably perceive a difference between a sample and its corresponding control, with 50% performance above chance. Wine is a complex matrix, and ODTs used in studies on wine can be based on inappropriate matrices and informal sensory methodologies. Formal studies confirming ODTs in wine are relatively scarce in the literature, and are complex and expensive to carry out. In this study, the sensitivity of panelists to previously published ODTs for five compounds: Guaiacol, *o*-cresol and 4-ethyl phenol, 3-isobutyl-2-methoxypyrazine (IBMP), and 2,4,6-trichloroanisole (TCA) associated with off-flavor/taint issues in wine, was investigated. The study was carried out in partially de-aromatized young Shiraz wine (unwooded) using a simplified version of the formal sensory approach. A triangle test in triplicate was carried out with 34 panelists, at the ODT for each compound, in one day. The study explored whether previous training affected panelists’ sensitivity for threshold differences. Results showed that samples spiked with volatile phenols were significantly different (*p* = 0.01) to controls. The spiked TCA and IBMP samples were not significantly different from the control in either case. Judges were better able to detect compounds if they had prior experience or training in wine evaluation. Despite some limitations, this pragmatic approach may be useful when carrying out sensory studies with fairly limited resources and within tight timelines, as it provides helpful information on panel members and detection thresholds for a specific matrix.

## 1. Introduction

Wine quality is difficult to define, but can be evaluated based on the sensory characteristics of the product using the sensory skills of experts, which include, but are not limited to, vision (clarity and color), gustation (taste), and olfaction (aroma). Wine aroma may be the most important factor in assigning quality, as faults, like microbial contamination and lack of typicality of style, are often detected by olfaction [1]. Frequently used indicators of the potency of aroma compounds associated with these faults include odor detection threshold (ODT) and odor activity value (OAV) measurements. Sensory thresholds are ill-defined in theory [2], but are based on the lowest concentration of a compound at which individuals can perceive a difference in sensory quality, relative to a control.

The ODT, which may be mg/L (ppm), µg/L (ppb), or ng/L (ppt), is numerically equal to the minimum concentration at which 50% of the judges succeed in differentiating a sample from a control [3]. The triangle test has been widely used for determining the detection threshold of volatile compounds important to wine aroma, including: Diacetyl [4]; oak lactone [5]; 3-isopropyl-2-methoxypyrazine [6], and rotundone [7]. The International Standards Organization (ISO) 13301 standard (encompassed in the American Society for Testing and Materials (ASTM) Standard Practice E679-04 [8]) has been used to establish detection thresholds for 2,4,6-trichloroanisole (TCA) [9] and ethyl hexanoate in solution with tannin and mucin [10]. The OAV is defined as the concentration of a compound present in a matrix divided by the ODT for that compound in that specific matrix. Generally, the larger the OAV, the more likely that compound would contribute to the overall odor of a complex odor mixture [10]. In theory, ODTs and OAVs may be determined this way for compounds of interest [10], but, in practice, a large number of issues influence the perception of these measures. Matrix effects, for example, have been shown to influence ODT perception in wine [11,12,13], and would therefore influence OAVs. The presence of other compounds in solution can also change the perception of target aromas, not just above the ODT (supra-threshold), but also below it (i.e., at sub- or infra-threshold). For example, the impact of infra- and supra-threshold concentrations of ethylphenols on wine was investigated [14], and it was found that both sub- and supraliminal concentrations of off-flavors not only changed the “hedonic valence” (positive or negative character) of the perception, but had a masking effect on fruity notes.

The composition of the panel used will also influence the success of sensory threshold studies. A panelist’s ability to detect an odor will vary depending on their level of fatigue, health status, and matrix effects in the study medium [8]. The reliability of the results can be increased by enlarging the panel and by replicating tests, but this will not negate matrix effects in another medium. Due to the unique nature of the product, sensory studies on aroma in wine are very specific to particular cultivars and styles, and frequently suffer from a lack of repeatability. For example, if research on the ODT of 4-ethylcatechol is reviewed, Larcher et al. [15] determined a detection threshold of between 100 and 400 µg/L in white and red wine, while another study determined a considerably higher odor threshold of 774 µg/L [16]. The Australian Wine Research Institute noted that the odor threshold of this compound increased to 1131 µg/L in “green” red wine, and 1528 µg/L in oaky red wine [16]. It would therefore seem obvious that ODTs are confirmed in the matrix in which a study is to be carried out, but evidence for confirmation is lacking in the literature.

In this study, five odor-active compounds were selected that had been associated with specific taint issues in wine aroma, namely guaiacol, *o*-cresol, 4-ethylphenol (4EP), 3-isobutyl-2-methoxypyrazine (IBMP), and 2,4,6-trichloroanisole (TCA). Aroma descriptors for these compounds cover a continuum from “burnt, smoky” and “medicinal” to the “moldy, dusty” flavors associated with cork taint (TCA), and “herbaceous, green” aromas associated with IBMP. All of these attributes have been associated with South African red wines [13,17,18,19,20]. In order to progress with work on interactions between these compounds in a partially de-aromatized red wine, it was necessary to confirm odor thresholds that had been previously determined (Table 1), and test sensitivity of potential panelists.

Guaiacol exhibited the lowest odor detection threshold of all the phenols tested in a red wine matrix by Boidron et al. [26]. It forms part of the “woody” family of descriptors [22], and is extracted from pyrolyzed (toasted) oak during wood maturation of wine. The detection threshold of 23 µg/L for guaiacol confirmed by Parker et al. [21] was chosen as it was determined in a relatively recent formal sensory study at the Australian Wine Research Institute in an Australian “base red wine”.

It is very difficult to find any sensory information in the primary literature about the cresols. There has been some work done in whisky, but the only current wine-related references concern its appearance in wine after smoke-events in the vineyard [27]. Boidron et al. [26] described *o*-cresol as “tar” (bitumen)-smelling, and gave an ODT of 0.8 mg/L. As the cited study was investigating wooded wines, and *o*-cresol is a known pyrolysis product of oak wood [28], this relatively high ODT seems reasonable. Parker et al. [22] reported a threshold, through a formal sensory process with 22 assessors, of 62 µg/L (standard error = 0.8) for *o*-cresol in “base red” wine. Due to the rubbery, tarry, and phenolic character of the compound, it was included in our study, and Parker’s [22] threshold used, as it is the only recent threshold available in the literature carried out in an unwooded base wine.

Associated with “brettiness” and “medicinal, phenolic” smells, as well as those of “leather/horse” and “bacon/meatiness”, 4EP has complex effects in wine. The detection threshold of 605 µg/L in red wine determined by Chatonnet et al. [23] is commonly cited. According to Escudero et al. [29], 4EP falls in the same semantic category as woody odorants, and in wooded wines, the wood character may mask the aroma character of the compound, making it more difficult to detect as a specific attribute (leather/horse/medicinal). This is in agreement with the findings of Curtin et al. [16], and seems to explain the high threshold found by Boidron et al. [26]. For the purposes of this study, Boidron’s published detection threshold was used, as it has been cited by a number of other researchers, and is based on recognized sensory methodology.

IBMP is a potent aroma-active compound often found at higher, above-thresholds concentrations that are detrimental to red wine quality [24,30] due to its “green/herbaceous/bell pepper” characteristic. Information on the determination of odor thresholds for pyrazines in the literature is limited. IBMP, specifically, is a very strong-smelling compound in its pure form, and its odor is pervasive and difficult to disperse. It is also known to cause skin, eye, and respiratory irritation [31]. Shibamoto [32] determined odor thresholds of 46 pyrazines in water, but did not test IBMP. French studies showed that IBMP was the main contributor to vegetal aroma in red Bordeaux and Loire wines from different vintages and cultivars [24], and the ODT was determined by comparing IBMP concentrations with the intensity of the green bell pepper character. Through this, the threshold value, which seems to be a recognition threshold, and not a perception threshold, was estimated to be 15 ng/L. This does not seem to have been confirmed by any further formal sensory studies.

TCA is a compound associated strongly with cork taint in wines and is described as having a “moldy” and “damp cardboard” odor. Tempere et al. [33] noted that amongst wine defects, 2,4,6-trichloroanisole has a specific impact on wine perception. In addition to giving to the wine an unpleasant odor, it has a strong masking effect on fruity notes. It is difficult to find information on formal sensory detection threshold determinations of TCA, but in a study on the effects of TCA on consumers’ wine perception [25], a level of 0.13 ng/L TCA was considered ”low” and 5 ng/L was considered “high” based on concentrations frequently found in contaminated red wines. TCA detection threshold depends on the wine organoleptic characteristics, and the person perceiving it, as wine professionals had very varying abilities towards TCA detection in wine. These authors tested detection of TCA with a panel selected based on their sensitivity to this compound, in a range of red and white wines, some of which were wooded. They found TCA was detected, in red wines, at 10–15 ng/L. Prescott et al. [34] established “consumer rejection thresholds” of TCA to be 3.1 ng/L and 3.7 ng/L in Chardonnay, depending on the panel. Although Mazzoleni et al. [9] found much higher levels for Italian red wines, and Cravero et al. [35] gave an identification threshold of 7 ng/L (in Barolo), the wine in this study was unwooded and partially de-aromatized, and therefore the detection threshold of 4 ng/L, as indicated by Prescott, was chosen as suitable for the matrix.

The formal procedures for ODT determination are outlined in standard methods, specifically ASTM E679 (Determination of Odor and Taste Thresholds By Ascending Forced-Choice (AFC) Concentration Series Method of Limits) [8]. Standard practice for defining and calculating individual and group sensory thresholds are outlined in ASTM E1432-04 [3]. Normally, individual threshold calculations would require 20 to 40 AFC presentations per panelist, with data sets taken at five or more concentration scale steps (typically six or seven), with pretesting to ensure individual thresholds fall within the testing range. Obviously, this is laborious, and as the ASTM standard itself states, the costs and availability of panelists places serious limitations on what can be covered by experimenters who, typically, are limited to panels of five to 15 individuals. Even with a limited panel, a simple threshold determination will involve 100–600 presentations, which would not be achievable for most researchers. This study looked at a simple, pragmatic approach to ODT sensitivity testing for the purposes of selecting panelists for a study.

From Table 1 and the discussion above, it is clear that ODTs found in water are not comparable to those determined in ethanol solution, and thresholds in white wine are different to those found in red wine. Differences between ODTs for various compounds within the categories of red wines (different cultivars [36], wooded/unwooded [29], for example) have also been shown to exist. Even on the rare occasions when thresholds are properly determined in wine according to the official E-679 method, they are affected by the specific wine matrix and style [11,37,38], and therefore do not necessarily have relevance in other matrices. It is also evident from the literature that different people have varying responses to compounds based on their culture, experience, and age, and that training may make a difference to a person’s ability to perceive an aroma [39,40,41].

For these reasons, the ODTs of compounds of interest to our work were investigated in a de-aromatized red wine matrix, which we planned to use in interaction studies. The sensitivity of potential panelist to compounds at ODT level was tested using a simplified version (three sets of samples per compound at the ODT) of the ASTM E-679-04 method. This study should thus provide an interesting perspective to researchers when they consider confirmation of ODTs in other matrices, as this is a pragmatic approach given the fairly complex and time-consuming standard practice. This research also set out to establish if panelists had particular sensitivities to compounds belonging to diverse aroma families, and whether previous training in sensory evaluation of wine influenced their ability to distinguish threshold differences between samples and controls.

## 2. Materials and Methods

### 2.1. Base Wine

A 2016 Shiraz wine (300 L) was supplied by a local wine producer (Koelenhof Cellar Ltd., Simonsberg, South Africa) and stored at 4 °C in 25 L food-grade plastic containers under nitrogen at the Department of Viticulture and Oenology, Stellenbosch University, South Africa. The wine had a pH of 3.6, and an alcohol concentration of 13% as determined by the supplier, who confirmed that the wine had not been treated with wood at any time during the winemaking process. Informal benchtop screening by five experienced sensory judges with tested sensitivity for the aroma compounds used to spike the wines confirmed that those compounds were not present in the base wine. The wine had an odor profile that was dominated strongly by fruit and berry aromas. This warranted partial de-aromatization following the method outlined by Wilson et al. (2017) [42] prior to threshold testing and investigations into sub-threshold interactions. The wine was de-aromatized by mixing thoroughly with activated charcoal powder (Merck, Darmstadt, Germany) for 12 h without agitation, then separated from the charcoal by diatomaceous earth filtration. During the treatment and blending steps, the wine was protected from oxidation under nitrogen gas. In a screening session, the expert panel chose a blend of 50:50 charcoal-treated wine to untreated wine, which yielded a neutral wine base with low aromatic intensity. Samples of the blend (50 mL) were taken to determine the baseline levels of the compounds investigated in this study. Analysis of volatile phenols in the de-aromatized wine was performed following the method outlined by De Vries et al. [43] using an Agilent Gas Chromatograph, model 6890N (Agilent, Palo Alto, CA, USA), coupled to an Agilent Mass Spectrometer 5975 B Inert XL EI/CI (Agilent, Palo Alto, CA, USA). Three technical repeats were analyzed for 13 volatile phenols (guaiacol; 2,6-dimethylphenol; 4-methylguaiacol; *o*-cresol; phenol; 4-ethylguaiacol; *m*-cresol; *p*-cresol; 2,3-dimethylphenol; eugenol; 4-ethylphenol; 4-vinylguaiacol; and 3,4-dimethylphenol). The base wine was found to contain very low or undetectable levels of any of the volatile phenols, including the phenols of interest. The guaiacol level in the base wine was 1.37 µg/L, *o*-cresol was 0.08 µg/L, and 4-ethylphenol concentration was 1.4 µg/L. The wine was also deemed, during informal tasting by the experienced sensory judges, to be completely free of any form of “moldy” or “herbaceous” odors that might have been associated with IBMP or TCA contamination.

### 2.2. Preparation of Spiked Wine Samples

The ODT levels chosen for the study were those that had been established by formal sensory methodologies, and/or were the most commonly used in literature for red wine (Table 1). These were 23 µg/L for guaiacol, 62 µg/L for *o*-cresol, 605 µg/L for 4EP, 15 ng/L for IBMP, and 4 ng/L for TCA. Stock solutions of 1000 mg/L of the five compounds were prepared in ethanol ≥ 99.8%, (Sigma-Aldrich, St. Louis, Missouri, United States). Guaiacol (99.3% purity), 4EP (99.5% purity), *o*-cresol (99%), IBMP, and TCA (also both 99%, Merck, Darmstadt, Germany. The compounds were dissolved in ethanol (10 mL) and then made up to volume with ultra-pure distilled water (Millipore, Bedford, MA, USA) to the concentrations required for spiking, i.e., 100 mg/L for *o*-cresol and guaiacol; 1000 mg/L for 4EP; 5 µg/L for IBMP; and 1 µg/L for TCA. Base wine (2.55 L) was then spiked with an appropriate volume of stock solution to achieve the concentrations of each volatile compound required for detection threshold determinations. For each compound, three sets of two control wines and one spiked was presented per judge. As there were 34 judges, 5.1 L of control wine was needed, and 2.55 L of spiked for each compound. Using a 100 mg/L stock solution of guaiacol, 230 µL were added to each liter of de-aromatized red wine to achieve a 23 µg/L concentration in the sample. The stock solution of *o*-cresol was also 100 mg/L, so 620 µL was used per liter of wine to achieve the 62 µg/L detection threshold level. 4EP was spiked with 0.605 mL of 1000 mg/L to reach 605 µg/L. The stock solution concentrations of IBMP and TCA were lower, and 3 mL and 4 mL of each was used to achieve a final concentration of 15 ng/L and 4 ng/L odor detection threshold levels for each compound, respectively. Base wine was spiked within 24 h of sensory analysis and stored at 5 °C in the dark. Stock solutions were stored at 5 °C in brown, sealed glass bottles, with the exception of the IBMP stock solution, which was stored at −20 °C in foil-wrapped containers to prevent light incursion.

### 2.3. Panel Selection

Thirty four participants (ages ranging from 21–54 years) were recruited from an existing pool of individuals who had previously volunteered for sensory evaluation testing in our facility, and were familiar with the format, task, and procedures involved in triangle testing. Panelists were screened against inclusion criteria comprising being of legal drinking age in South Africa and consuming wine at least once every six months. Exclusion criteria comprised being a smoker and having taste or smell defects. The panel consisted of four males and 30 females of varying abilities from novices to very experienced wine tasters. All participants provided their informed consent before participating in the study. All sensory data was obtained in compliance with institutional procedures for sensory evaluation (Ethical Clearance VIT-2018-6570). For the purposes of this study, “experienced” judges (*n* = 18) were those who had been members of wine sensory panels within the university and/or the wine industry for at least six months. “Inexperienced” judges (*n* = 16) were not members of sensory panels, and although they indicated they regularly consumed wine, most had no formal wine sensory training.

### 2.4. Sensory Testing

A triangle test was carried out to test the sensitivity of panelists for the selected compounds (three volatile phenols, TCA, and IBMP) in red wine. As this was not a full detection threshold determination, the investigation was carried out using a simplified version of the triangle test method [8]. Sensory evaluation of the wines was conducted in a sensory laboratory equipped with individual booths with standard artificial daylight lighting and temperature control at 20 ± 1 °C. One hour before serving, wines were equilibrated to room temperature before being poured into black ISO 3591:1977 standard glasses and covered with plastic lids. Each panelist worked in a white isolated booth and no communication was permitted between panelists. Testing for all five compounds was conducted on the same day, in triplicate. Participants therefore received five sets of 30 mL samples, one for each compound of interest. Each set contained brackets of two control (clean) wines, and one wine which had been spiked with one of five compounds under investigation. The samples were labelled with individual three digit codes and presented in a randomized order per panelist as recommended by Lawless and Heymann [2]. Panelists only evaluated samples orthonasally, as the study was concerned only with odor thresholds and aroma effects, and not with palate effects. From each set, panelists were asked to select the odd (or different) sample. Judges tested the compound ODT in the following order: Guaiacol, *o*-cresol, 4EP, IBMP, and TCA. To cancel strong carry-over effects and to minimize tiredness of the sense organs, judges were asked to rest for fifteen minutes between sets. Results of the triangle test were then examined to determine whether judges’ experience made a difference to their abilities to correctly identify spiked samples. Panelists were deemed sufficiently sensitive if two out of three replicates were correct.

### 2.5. Data Analysis

In each set, if a judge had 0 or 1 out of three brackets correct, the overall result for the judge was considered a “0” or incorrect (not detected). If the judge had two or three correct brackets, the judge was considered a “1” or correct (spiked sample detected). Results were counted to determine whether 50% or more of the judges had detected samples spiked at the detection threshold. Counts were compared to the minimum number of correct judgments required for significance at probabilities of 5% and 1% for the triangle test (one-tailed) [26]. The data were separated into “experienced” (*n* = 18) and “inexperienced” judge (*n* = 16) groupings and any differences between groupings during the ODT investigated. This raw “judge response” data was further analyzed post hoc to investigate perceptual relationships between compounds and categorical factors using Excel 2017 (Microsoft Office, Redmond, WA, USA) and Statistica 12. The additional analyses were carried out using mixed model ANOVA. Least Squares Means (LSM) were generated for the panelists and per compound using this data. When a significant effect was observed, the Fisher’s Least Significant Difference (LSD) test was used to compute pairwise comparisons with α = 0.05. The quantitative factors were analyzed by Spearman’s and Pearson’s correlations.

## 3. Results

### 3.1. Investigation of Odor Detection Thresholds

**Figure 1:**
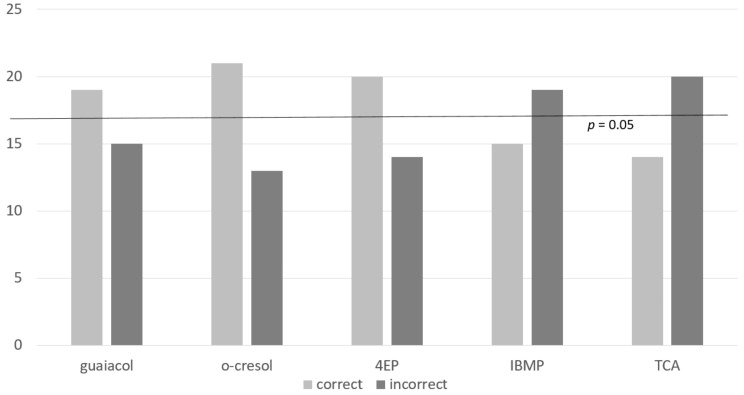
Correct vs incorrect judgments per compound (*n* = 34 judges).

**Figure 2:**
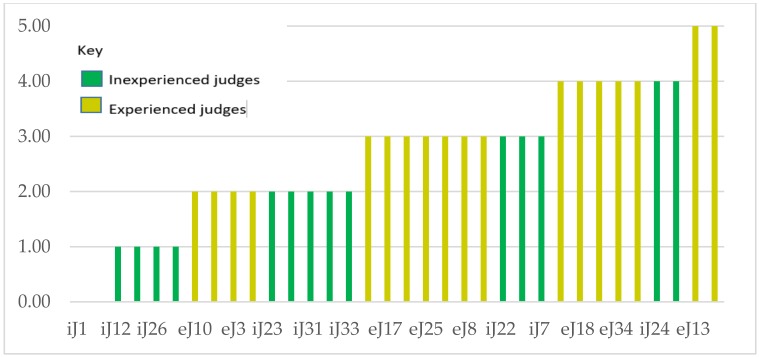
Number of correct judgements per experienced (ej) and inexperienced judge (ij) by 3-AFC Triangle Test for five compounds.

**Figure 3:**
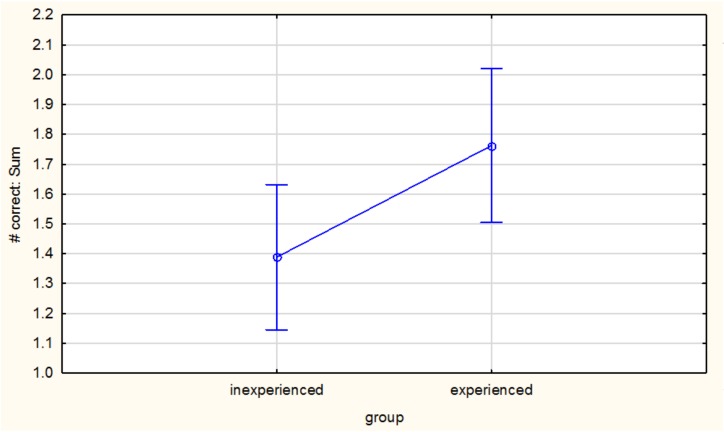
Group effect (Least Squares Means) of experienced vs inexperienced judges (Vertical bars denote 0.95 confidence intervals).

**Figure 4:**
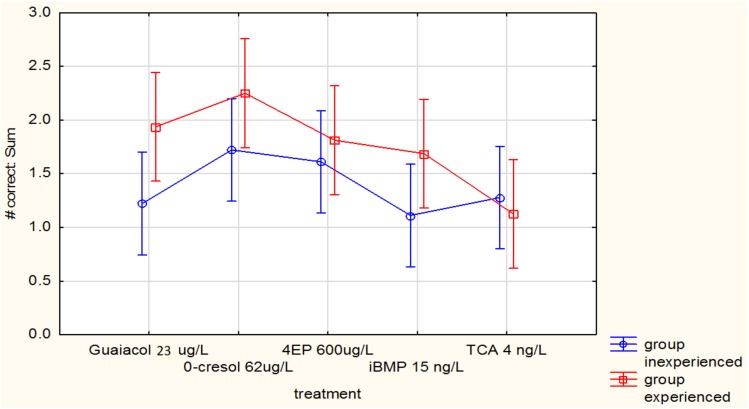
Number of correct judgements per experienced and inexperienced judge by 3-AFC Triangle Test (LS means) for individual compounds. Vertical bars denote 0.95 confidence intervals.

To confirm an odor detection threshold using the standard sensory methodology, the number of judges selecting the different (spiked) samples correctly must be at or around 50% of the participants [8]. Roessler tables [44] indicate that if 34 judges are used to carry out the triangle test (as in this case), results are significantly different from the control at the 5% level (*p* = 0.05) if 17/34 judges make the correct choice over the complete set for a compound (i.e., 50% of the participants). If 19/34 judges choose correctly, the results are significant at the 1% level (*p* = 0.01). Results are presented in Figure 1, Figure 2, Figure 3 and Figure 4. None of the compounds were therefore at exactly their ODTs for this matrix. As can be seen in Figure 1, *o*-cresol was most easily detected at its ODT, 4EP and guaiacol were also successfully detected by a significant number of judges, and TCA and IBMP were less easily detected in this matrix.

In this study, the de-aromatized Shiraz wine was spiked with the equivalent of 23 µg/L of guaiacol. On a per judge (*n* = 34) basis, taking two or more responses as correct per triad, 19 judges chose correctly, and 15 chose incorrectly. This again is significant at *p* = 0.01 [44]. This indicated that guaiacol, spiked in de-aromatized Shiraz at a concentration of 23 µg/L, was detected by the majority of tasters and therefore the ODT was confirmed.

For the second volatile phenol in this study, 21 of the 34 judges were able to detect *o*-cresol at this level (62 µg/L) in de-aromatized Shiraz wine. This result is significant at *p* = 0.01 level. This means that a spiking level of 62 µg/L is higher than the ODT for *o*-cresol in this matrix. Polyphenols and ethanol have both been shown to have an effect on the perception of volatile phenols [37], and with other aroma compounds removed, the *o*-cresol may have been more obvious to the judges in this study than those involved in previous studies with fully aromatic red wine.

Very similar results to those of the other two volatile phenols were obtained for 4EP. Twenty (59%) of the 34 judges were able to detect a difference between samples spiked with threshold levels of 4EP in a de-aromatized Shiraz. This result was significant for the triangle test at *p* = 0.01.

In this study, the detection threshold of 15 ng/L for IBMP was chosen, because although Roujou de Boubée et al. [24] found higher levels in some French red wines, the wine in this study was unwooded and partially de-aromatized, and it was felt that a higher concentration might be too easily detected. Despite this, participants had difficulty detecting IBMP in the de-aromatized Shiraz matrix at this level. Only 15 (44%) of the 34 judges were able to detect a difference between samples which was not significant. Van Wyngaard et al. [45] showed that IBMP had an interactive effect on thiols in solution in Sauvignon blanc. Although there is not a lot of research on the masking effect of IBMP, other research [34] has shown, using canonical variate analysis, that wines spiked with either bell pepper or fruit were separated on the fruit/bell pepper continuum, i.e., that a masking effect of vegetative aromas by fruit aromas occurred. It may be the case in this study that the fruity aromas of the Shiraz masked the vegetative green pepper aroma for the less experienced judges, or that these judges felt sensory fatigue.

Participants also had difficulty detecting TCA in the de-aromatized Shiraz matrix. Only 14 judges (41%) of the 34 judges were able to detect a difference between controls and samples spiked with threshold levels of TCA at the 4 ng/L level. This finding substantiates others [9] that higher levels are needed in a red wine matrix as it is more complex. TCA is known to be a particularly “sticky” compound [40] with a potent inhibitory effect on olfactory cell responses, showing slow kinetics with an integration time of approximately 1 s. Even at infra-threshold levels, TCA has a marked masking effect on other odors [25], and these effects are sustained. It is also well established that judges vary substantially in their abilities to detect TCA: from 1 to 250 ng/L amongst experienced panelists, and from 2.5 ng/L to 25,000 ng/L in inexperienced judges [46]. As in the case of IBMP, it is possible that judges experienced sensory fatigue by this stage of the testing, and masking effects of other compounds may have decreased sensitivity of panelists to TCA. However, the ODTs of IBMP and TCA may be at higher concentrations in de-aromatized Shiraz, and an additional study, with increased concentrations of both compounds, would be useful to confirm this. The judge responses in detection of volatile phenols, and the latter two compounds show that although a single level provides some information regarding the ODT in a specific matrix, to truly establish the ODT for a compound, it is necessary to carry out a full sensory study as recommended by ASTM 679-04 [8].

### 3.2. Differences in Frequency of Correct Detection between Compounds by Panelists

Table 2 shows the results for Pearson’s (linear relationship) and Spearman correlation (monotonic: Whether linear or not) coefficients between compounds in solution, with −1.0 indicating a perfect negative correlation, and 1.0 indicating a perfect positive correlation. Values for relationships varied between −0.13 and 0.27, indicating there are only very weak positive and negative correlations between some of the variables. This is substantiated by the *p* values, none of which are below 0.11, an indication that relationships that do exist are not significant. The results of the two analyses therefore gave the same outcomes.

In a second attempt to identify any relationships between compounds, the number of correct selections within the three repeats were added, giving a score between 0–3, and this was analyzed in the standard way using mixed model ANOVA (Table 3). A generalized estimating equations (GEE) analysis on the raw 0/1 outcomes was carried out. The GEE method is often used to analyze longitudinal and other correlated response data, especially if responses are binary [47], as in this comparison.

In this case, most pairs of compounds showed no significant differences in detection by panelists, but there were two exceptions. Judges who detected *o*-cresol seemed to have been able to detect IBMP and TCA more readily and there was also a weak tendency for judges who detected 4EP to be able to detect TCA more readily. This would need to be tested with a more directed study looking at different levels of compounds before any assumptions about these relationships could be made. 

For the purposes of further exploring relationships and panelists’ responses, compounds were grouped into aroma families according to Noble et al. [48]. The volatile phenols (guaiacol, 4EP, and *o*-cresol) encompassed a group of descriptors that fell into the “woody” category, and IBMP into “vegetative” and TCA into “earthy”. There did appear to be significant differences in panelists’ abilities to detect between odor families. There was a definite predominance (21 of the 34 judges, or 62%) of correct choices associated with the phenolic “woody” family of compounds across both groups (experienced as well as inexperienced panelists). The TCA/IBMP group predominated in correct identifications in only eight (23%) of the judges, and in five (15%) judges’ cases, there was no predominance of either aroma family.

The hedonic valence of an odor has been shown to influence its perception by panelists [41]. The predominance of correct identification associated with volatile phenols may be because humans generally associate woody, burnt smells with cooking/food and home fires, and therefore prefer and detect them. It could also be that judges unconsciously avoided selecting samples with moldy or unripe (green) characters, as the ability to be put off unsuitable/unhealthy food before tasting or ingesting it may confer an evolutionary advantage through infectious disease [49]. Another explanation may be that panelists have different sensitivities for different groupings of compounds based on their cultural backgrounds, as found by previous works [39,40]. These suppositions in the context of this study need to be tested with further research.

### 3.3. The Impact of Previous Training or Exposure on Judge Sensitivity

Tempere et al. [50] noted that even wine tasting “experts” show high olfactory detection thresholds for key compounds of wine, which is not ideal when wine quality depends on fault-detection at low levels. Kaeppler and Mueller [41] raised the question whether olfactory systems may be in fact linguistic arrangements based on experience and exposure to expert lexicons. Whether odor categories are innate or learned depends on the influence of language on odor processing. To investigate whether previous training or exposure of a participant (for example, as a member of a sensory panel) impacted sensitivity to threshold differences between samples and controls, panelists in this study were separated into “experienced” and “inexperienced” groups, and differences in their responses/results during the sensitivity study were investigated.

Figure 2 shows the results for individual judges, and their abilities to detect differences between spiked and control samples. Out of 34 panelists in our trial, five did not detect guaiacol at all, four could not detect *o*-cresol, and six judges of the 34 (not the same people) were unable to detect 4EP, TCA, and IBMP. Out of the 34, there were 15 judges who detected all the compounds, and two judges who were unable to correctly identify any of the spiked samples. The average number of compounds correctly identified by experienced judges was 3.69, with a median of 4, and an SD of 0.7. Inexperienced judges, on the other hand, had an average for correct judgments of only 1.72, with a median of 2.00 (SD 0.89). In the inexperienced group, none of the judges were able to perceive all five compounds in spiked solutions. It is obvious, therefore, that training and experience must have sensitized the experienced judges to the very subtle differences between wines.

The Least Squares Means (LSM), generated using the mixed model ANOVAs for inexperienced vs experienced judges (*p* = 0.04) indicate that the overall group effect is significant (Figure 3); experienced judges were better able to correctly identify spike samples from controls. This could be due to higher smell acuity generally or through experience working on wine evaluation panels. In other circumstances (for example, research project panels that require a particular acuity or sensitivity for a specific compound), it may have been advisable to exclude judges with poor or inconsistent performance, and/or omitting their observations from the data set. This concurs with what other researchers have recommended [14], that adapted training for professionals in the wine industry will enhance abilities of judges to differentiate between spiked samples and controls, even at ODT levels.

Figure 4 indicates averages of judgements for each compound (out of three) across all the judges. Only trends can be seen, for example, experienced judges seemed better able to detect the spiked samples in the case of guaiacol, *o*-cresol, and IBMP, but these differences were not significant (*p* = 0.38). There was no difference at all between judges, whether experienced or inexperienced in detecting the spiked TCA samples, and little difference in the case of 4EP. As IBMP and TCA are the two compounds that appear to show the lowest number of correct judgments, it is quite probable that the assessors also experienced sensory fatigue. Ideally, these compounds should have been tested on different days.

## 4. Conclusions

To explore a pragmatic approach to judge sensitivity for five taint compounds (guaiacol, *o*-cresol, 4EP, TCA, and IBMP) in red wine, three triangle tests (two controls, one sample spiked at the selected ODT for each compound) were carried out for 34 judges. Spiked samples were correctly differentiated from controls in the following order: *o*-cresol (62% correct), 4EP (59% correct), guaiacol (56% correct), TCA (44% correct), and IBMP (41% correct). What could be deduced from an analysis of correct identification of spiked compounds was that correlations in perception between pairs of compounds was very weak. If a particular compound was detected by the judges, it did not have a bearing on another group, but judges that had higher scores generally were more likely to detect all compounds across both families. As IBMP and TCA were the last two compounds to be assessed of the five, and also the two compounds that showed the least correct responses or differences between experienced and inexperienced judges, it is probable that the assessors experienced sensory fatigue. This is a limitation of this strategy, and if the study were to be repeated, it would be advisable for each compound to be tested on a different day at different levels, or presented in a random order to the judges. Ideally, a full ODT study should be carried out to determine ODTs for a specific matrix, but the simplified method will be a frame of reference for further work. In this study, the responses of the judges confirmed that the ODTs for guaiacol, and 4-ethylphenol in de-aromatized Shiraz wine, but an additional, more comprehensive sensory study to establish whether the levels could be reduced would be useful to determine the actual thresholds of detection in this matrix.

The concentration of *o*-cresol could certainly be reduced in future work in this matrix, as almost two thirds of the panelists were able to detect spiked samples, and concentrations of IBMP and TCA could be increased, as only 44% and 41% of judges could distinguish these compounds spiked at accepted ODT levels in this matrix. This may also have been due to sensory fatigue, and is a limitation of testing this number of compounds on one day.

One interesting aspect that emerged from this work was the ability of judges to detect compounds more easily if they had some experience of winetasting, showing that training can possibly sensitize people to attributes that they might not have noticed previously. The confirmation of ODTs in a partially de-aromatized red wine matrix has opened the way for further research and the possibility of training industry members to sensitize them to these off-flavors. The unique and complex interactions between language, training, and olfaction should definitely be assessed in future studies. This study highlights how important it is to understand the detection thresholds of compounds in different products, and has relevance for many foods and beverages where sensory thresholds from the literature may be taken for granted and not tested in the study matrix.

## Figures and Tables

**Table 1:** Odor Detection Thresholds (µg/L) and descriptors for compounds in red wine.

Compound	Odor Detection Threshold	Descriptors	Reference
**Guaiacol**	23 µg/L	Burnt, smoky, toasty phenolic	[21]
***o*-Cresol**	62 µg/L	Burnt smoky, medicinal, tar	[22]
**4EP**	605 µg/L	Leather, bacon, medicinal, horse	[23]
**IBMP**	15 ng/L	Bell pepper, green, herbaceous	[24]
**TCA**	4 ng/L	Moldy, musty, damp cardboard	[25]

**Table 2:** Correlation strength between compounds according to Pearson and Spearman correlation coefficients of correct identification of spiked samples by tasters between compound pairs.

Triangle Test Correlations between Compounds (*n* = 34)					
Compound 1	Compound 2	Pearson	Pearson *p*	Spearman	Spearman *p*
*o*-Cresol	4EP	−0.06	0.74	−0.05	0.78
*o*-Cresol	Guaiacol	−0.13	0.46	−0.10	0.56
*o*-Cresol	IBMP	0.19	0.28	0.19	0.27
*o*-Cresol	TCA	0.06	0.75	0.04	0.82
4EP	Guaiacol	0.08	0.65	0.06	0.74
4EP	IBMP	0.00	0.98	0.03	0.88
4EP	TCA	0.28	0.11	0.27	0.12
Guaiacol	IBMP	0.24	0.17	0.24	0.17
Guaiacol	TCA	0.16	0.37	0.14	0.42
IBMP	TCA	0.01	0.96	0.02	0.91

**Table 3:** Least Significant Difference comparisons between compound pairs.

Comparisons	1st Mean	2nd Mean	Mean Diff	*p*
1-2	Guaiacol	*o*-Cresol	−0.41	0.09
1-3	Guaiacol	4EP	−0.15	0.54
1-4	Guaiacol	IBMP	0.18	0.47
1-5	Guaiacol	TCA	0.35	0.15
2-3	*o*-Cresol	4EP	0.26	0.28
2-4	*o*-Cresol	IBMP	0.59	0.02
2-5	*o*-Cresol	TCA	0.76	0.00
3-4	4EP	IBMP	0.32	0.18
3-5	4EP	TCA	0.50	0.04
4-5	IBMP	TCA	0.18	0.47
